# Explaining the Unexplainable: Balancing Responsibility, Expectations, and Identity in Narratives of Sexual Recidivism

**DOI:** 10.1177/10790632241268478

**Published:** 2024-07-25

**Authors:** Ingeborg Jenssen Sandbukt

**Affiliations:** 1 Department of Mental Health and Addiction, Centre for Research and Education in Forensic Psychiatry, 155272 Oslo University Hospital, Oslo, Norway; 2 Department of Criminology and Sociology of Law, University of Oslo, Oslo, Norway

**Keywords:** sexual reoffending, sexual recidivism, narrative criminology, desistance

## Abstract

When someone is caught and punished for a sexual offense, recidivism prevention is of high priority. While a growing body of qualitative research has investigated the desistance process of those who have sexually offended, few studies have examined the narratives of individuals who have sexually recidivated in order to understand how they make sense of their reoffending. This study aims to fill this gap in the literature by exploring the recidivism process and broader life stories of 16 imprisoned men through semi-structured interviews. The results demonstrate how these men explain their recent “failures,” the obstacles they face when doing so, and how they attempt to overcome these obstacles in their narratives. Thus, the analysis in this study is both thematic and narrative. Finally, the findings’ practical implications are discussed to show how ambiguity in narratives can be a powerful tool in correctional and clinical interventions.

Sexual crimes evoke fear, anger, and negative emotions (e.g., Harper et al., 2017; Zatkin et al., 2021). Although sexual recidivism is statistically rare (Hanson & Bussière, 1998; Hanson & Morton-Bourgon, 2005), the associated human, economic, and societal consequences of it are severe (Peterson et al., 2017). Therefore, this phenomenon has received considerable scholarly attention. Researchers have investigated risk factors, useful ways to assess levels of risk, and the long-term effects of correctional and therapeutic interventions aimed at preventing sexual recidivism (Gannon et al., 2019; Helmus et al., 2012; Schmucker & Losel, 2015). However, sexual recidivism is rarely studied from the perspective of those who recidivate after a sexual offense conviction, much less how they account for their reoffending.

Behind every incident of sexual recidivism, there is a story, or perhaps several stories. However, the recidivism process poorly understood. Most qualitative studies of individuals with multiple sexual offense convictions have tried to understand the desistance process. Many of these studies have adopted a narrative approach, investigating how individuals with a criminal past explain, in story form, how they eventually managed to *stop* offending (e.g., Harris, 2017). While desistance stories represent success, recidivism represents failure—the fact that rehabilitation did not work and that the individual failed to desist. Although desistance stories involve an account for previous (re)offending, they are told in hindsight—when the narrator identifies as a desister and can explain the cognitive transformation process that has supported the creation of a “new me” or a “real me” (Maruna, 2001). The stories told when these self-narratives are not yet established may be equally important as they can increase our understanding of sexual recidivism. These stories are the focus of this article. Sixteen imprisoned men with a history of sexual recidivism talk about how and why they reoffended. The aim is to demonstrate how these men account for their failures. Furthermore, by describing the obstacles they face in providing these accounts and how they attempt to overcome them, I aim to shed light on the purposes served by their narratives.

## Background

### Defining Sexual Recidivism

According to Francis and Soothill’s (2014) definition, *sexual recidivism* is concerned with the sexual reoffending of individuals who have already had contact with the criminal justice system for a sexual offense. In practice, however, individuals can sexually offend as well as reoffend without being caught, prosecuted, or convicted. This leaves the actual rate of sexual recidivism unknown (Lussier et al., 2021). Over the past 70 years, a large body of quantitative research has been produced in the sexual recidivism field (Lussier et al., 2023). Despite differing operationalizations of recidivism (e.g., new arrest or new conviction), this research has shown that rates of sexual recidivism are low compared to those of other crime types and that the likelihood of new sexual offenses declines in proportion to the amount of time that the individual remains free of sexual crime in the community (Hanson et al., 2018). Meta-analyses indicate that 10%–15% of individuals sentenced for a sexual offense receive a new conviction for a subsequent sexual crime within five years (Hanson & Bussière, 1998; Hanson & Morton-Bourgon, 2005). A Norwegian study showed that less than 4% of individuals released from a sexual offense sentence served in prison received a new sexual crime conviction within a mean follow-up time of six years (Sandbukt et al., 2021). However, despite generally low recidivism rates, some people pose a higher risk than others do. Past criminal behavior is among the best predictors of sexual recidivism (Hanson & Bussière, 1998; Hanson & Morton-Bourgon, 2005). Individuals with a history of sexual offending are statistically more likely to reoffend; for this reason, they are often targeted for interventions while imprisoned. Intervention type and intensity can vary, but practices that adhere to the risk-need-responsivity (RNR) principles (Bonta & Andrews, 2017) have generally been found to be most effective in reducing recidivism risk.

### Desistance From Sexual Offending: A Complex and Related Process

While *desistance* can be conceptualized in a number of ways, it is generally taken to refer to the dynamic and often complex process through which individuals who have previously been engaged in patterns of offending refrain from and/or decrease these behaviors over time (Kazemian, 2007; McAlinden et al., 2017). This process has been divided into three stages or phases. A primary and secondary phase, distinguishing simple behavioral change (on its own) from an accompanied shift in the individual’s identity, was conceptualized by Maruna and Farrall (2004). Later, to acknowledge the fact that desistance is a social process as much as a personal one, McNeill (2014) suggested a tertiary phase involving a sense of belonging to a (moral) community. There is now wide agreement that, in general, desistance is not something that just “happens.” Two overarching sets of explanations have emerged to account for this behavioral change. The first one concerns the presence of informal social control instances (e.g., relationships, employment, and meaningful social activities) over the life-course (e.g. Sampson & Laub, 1993). Such explanations suggest that social bonds have the potential to support the desistance process by offering structure and a sense of community and belonging. The second set of explanations concerns changes in the way people with a history of offending think about themselves and their opportunities, also referred to as cognitive transformation or identity change (Giordano et al., 2002; Maruna, 2001). Key themes here are the importance of the reflective self, hope, and the ability to imagine a plausible new personal identity. Thanks to these elements, the individual develops a sense of agency whereby he realizes that he has the potential to change his behavior and influence his future (Maruna, 2001). Previous studies have suggested that this process begins in the early stages of desistance (King, 2013; McAlinden et al., 2017; Ugelvik, 2021). Although some scholars suggest that desistance is not dependent on the presence of prosocial influences (Lussier & McCuish, 2016), social recognition has been found to be a key driver of this identity change, which means that the two sets of explanations are complementary (Weaver & McNeill, 2014). These findings on desistance as a general criminological phenomenon mostly apply also to people who desist from sexual offending, though there are some notable differences (McAlinden et al., 2017).

Although desistance concerns the ceasing of criminal behavior (what we want) and recidivism traditionally represents the continuation of criminal behavior (what we fear), the two are related concepts as they constitute important elements of a criminal career. The majority of qualitative studies within this field has however so far either focused on the factors that have hindered desistance or been limited to samples who have identified as desisters and are recruited on this basis. Wakeling (2022) interviewed six men who had attended treatment while imprisoned and had later reoffended sexually. Her findings indicated that despite being hopeful, determined to stay out of prison, and positively inclined toward the future before release, “this positivity was diminished over time due to other aspects of their life on release, such as difficulties with employment, housing and healthcare” (p. 54). Harris (2017) interviewed men with several sexual offense convictions about their life stories in general and their desistance processes in particular. Some of the men shared stories about their relapse experiences, though this was not the main focus of the study. In these narratives, three main themes kept emerging: (1) relapse occurred soon after release; (2) the presence of resources upon release created a false sense of security; and (3) in hindsight, in their opinion, these individuals were not ready for release, even though they had thought they were at the time (Harris, 2017, p. 112). In the present, however, Harris’ interviewees *identified* as “desisters” and their stories thus accounted for how their “desistance was interrupted” (p. 110).

While most desistance studies recognize that reoffending can occur on the path to a crime-free life, recidivism is often “narratively reduced” to a hindrance along the desistance journey. In fact, the recidivism process *per se* is rarely explored in detail. This is the first time that a narrative lens is being applied to explanations of recidivism among individuals who have reoffended sexually, are still incarcerated, and who are recruited for this exact reason.

### Narrative Criminology, Narrative Identity, and the Power of Stories

Narrative criminology is “a theoretical paradigm centered on the view that stories influence human actions and arrangements, including those that harm” (Presser & Sandberg, 2019, p. 131). The study of desistance is undoubtedly the area in which narrative criminology has had the most sustained development (Maruna & Liem, 2021). In his book *Making good*, Maruna (2001) described how a redemption script often characterized the narratives of the desisters in his sample. By reaching back into early experiences and “rewriting their shameful pasts” (p. 87), these individuals managed to create a coherent and prosocial identity in story form. A story or narrative, then, is not simply a rendering, report, or record of what happened; rather, it is a selection of lived experiences and an attempt at meaning-making (Presser, 2009; Presser & Sandberg, 2015). Studying narratives means paying particular attention to the subjectivity of storytelling and the functions self-narratives serve in identity work (McAdams, 1993). According to McAdams and McLean (2013), stories have the power to create a narrative identity through which “people convey to themselves and to others who they are now, how they came to be, and where they think their lives may be going in the future” (p. 233). Moreover, stories are socially constructed and dynamic entities. Through stimulation, new narratives may become available and reshape the overall stories people tell about themselves and their lives. This notion is the cornerstone of narrative therapy; one of several therapeutic strands that see identity as a relational process (Freedman & Coombs, 1996). Narrative therapy as well as narrative criminology recognize that stories can shape future action in the sense that people will, perhaps both consciously and unconsciously, strive to live up to the identity they verbally create (Presser & Sandberg, 2015). In other words, life stories and thus identities can be re-*authored*.

Relatedly, stories can be “plurivocal” or “elastic” in the sense that they are constructed and told differently in different contexts and to different audiences (Maruna & Liem, 2021; Presser, 2004; Sandberg et al., 2015). Warr (2020) studied the narratives of indeterminately sentenced and now incarcerated individuals in the United Kingdom. Due to their uncertain situations and futures, these individuals knew that they needed to convince others that they had changed in order to be considered for release. Warr referred to their demanding task of creating narrative acceptance in others by portraying themselves in certain ways and saying the right thing as “narrative labour” (p. 30). This concept seems applicable to those serving sentences for repeated sexual offenses. While imprisoned, these individuals are expected to take responsibility for their actions and demonstrate an ability and willingness to make prosocial changes in their lives. Furthermore, despite broad critiques, treatment programs aimed at preventing subsequent offending often aim to eliminate the use of excuses and so-called “cognitive distortions” (Friestad, 2012; Marshall et al., 2001). However, several studies have found that people will attempt to excuse different types of offensive behavior by searching for external, unstable, and specific causes rather than internalize personal responsibility (see Maruna and Mann [2006] for an overview). Excuse-making and the justification or neutralization (Sykes & Matza, 1957) of offensive behavior are not in themselves criminogenic; in other settings, they are viewed as normal, healthy, and socially rewarded behaviors (Maruna & Mann, 2006). A few studies have looked into these mechanisms in the accounts of individuals who have sexually offended. For instance, Farmer et al. (2012) compared a group of male desisters to a group of men who were potentially still active in sexual offending. Their findings indicated that the potentially still active individuals were less agentic than the desisting group and more likely to place blame on external factors and feel alienated and isolated from the community. This indicates that an alteration of self-narrative may be key to the cognitive transformation that desisters often demonstrate through redemption scripts (Maruna, 2001).

Storying desistance, or rewriting the past into a desistance narrative, involves explaining not only why one did what one did but also why one is not really like that, or no longer like that. Hence, in a desistance narrative, personal growth is enabled (McAdams & McLean, 2013). The fact that desisters describe their criminal careers and cognitive transformations in retrospect provide them with a narrative advantage. The story, and hence the narrator, can focus less on the failure and more on the outcome—the “future self” or “real me” that does not reoffend. Also, excuses might no longer be seen as excuses but as highly relevant explanations contributing to the individual’s insight and subsequent change. This is exactly why the failure accounts of those who identify or appear as desisters might not be applicable in the case of all imprisoned individuals with a longer criminal history. Desistance is only one dimension of a criminal career (Kazemian, 2007) and the situations of those who are still working toward a crime-free future are different in several ways, especially in terms of social context and expectations.

### The Current Study

Given the gaps in our understanding of the criminal careers of imprisoned individuals with multiple sexual offense convictions, the current study aimed to investigate the recidivism narratives of this population. The aim was not to uncover “the truth” about the participants’ reoffending or decide whether their stories were realistic. Instead, my aim was to explore how men who have sexually reoffended accounted for their failure. In particular, I explored how the men used the narratives “available” to them while attempting to balance responsibility, expectations, and identity in their accounts of recidivism.

## Method

### Context

The findings presented in this article come from a qualitative study of men convicted and incarcerated in Norwegian prisons for sexual offenses committed after release from prior sexual offense convictions. Norway is recognized globally for its “exceptional” approach to crime and punishment, demonstrated through low imprisonment rates, humane prison conditions and generally inclusionary penal practices (Pratt, 2008). Furthermore, due to the absence of registration requirements and notification laws for individuals convicted of sexual crimes, structural stigma is less pronounced in the country. However, social stigma toward this group is present (Sandbukt, 2023).

When the recruitment for this study began (March 2021), approximately 700 individuals were serving sentences in Norwegian prisons for sexual offenses (Statistics Norway, 2021). About 80 of these men had at least one prior prison sentence for a sexual offense. Out of these 80, many were serving *forvaring* (the Norwegian word for an indeterminate sentence similar to “preventive detention” or “civil commitment”). Although most individuals will eventually be released, a *forvaring* sentence allows for the lifelong detention of a person if they continue to pose a danger to society. For these individuals, psychological treatment offers an opportunity to work on their risk factors and progress toward conditional release. Those sentenced to *forvaring* are usually held in designated units, though they are not separated by crime category. Unlike many other jurisdictions, those serving fixed sexual offense sentences most often serve their time in general population units, and their access to psychological treatment and programs vary depending on their assumed risk levels, the length of their sentences, and what the units offer. Treatment is not mandatory for incarcerated individuals in Norway, including those convicted of sexual crimes, and access to services have been limited. Only recently have individuals who pose a higher risk of sexual reoffending systematically been identified and offered specialized psychological treatment in Norwegian prisons.

### Recruitment

Recruitment was facilitated by Correctional Service contact persons in each eligible prison unit. I informed the contact persons about the study’s purpose and main inclusion criteria (multiple sexual offense sentences); they then independently assessed which eligible individuals were mentally fit (e.g., not suffering from psychosis) and could thus participate. As data collection relied on in-person interviews, an additional inclusion criterion was the ability to communicate in Norwegian. The contact persons approached each potential participant, providing them with an information sheet that explained the study’s purpose and what taking part in it would entail. The information sheet emphasized the voluntary nature of the study; it also warned potential participants that the interviews would include questions about their broader lives, not only their criminal careers, and that some topics could be experienced as emotionally challenging. Those who agreed to participate were then scheduled for an interview. No identifiable information about them was available to me before they had consented to participate. All the information about the participants’ lives, demographics, and criminal histories (other than the fact that they had multiple sexual offense convictions) was self-reported. Recruitment ended after approximately one year, after the inclusion of 25 men who fulfilled the criteria for being considered recidivists, and when the number of inquiries from prison contact persons stopped due to a lack of new individuals who met the inclusion criteria. Two men later decided to withdraw; thus, the final number of participants in the overarching qualitative study were 23.

### The Sample

The analysis presented in this article is based on a subsample of 16 men; six interviewees were excluded as they denied (to various degrees) having reoffended despite having been reconvicted. Although denial is common and can serve several self-upholding functions (Blagden et al., 2014), these men were ultimately excluded from the current study because its purpose was to investigate how the men accounted for their reoffending. The participants have been assigned pseudonyms.

The 16 men included varied in age from 30 to 66, with a mean age of 46. At the time of the interviews, they had been incarcerated for their index offenses for between one and 10 years. The majority (*n* = 13) were serving *forvaring* sentences, whereas the rest (*n* = 3) were serving fixed sentences ranging from five to 12 years. A small number had been convicted of adult rape, while the majority was convicted of child sexual crimes (contact, non-contact/online, or both). The number of previous sexual convictions ranged from one to four. The severity and nature of their crimes differed, as did the length of their known periods of activity in terms of offending. Their self-reported time to reoffending varied between a few hours and two decades after the previous release from prison. While most of the men had some kind of treatment or program experience from prison or the community, the intensity, format and duration of these varied.

### Data Collection

Two in-depth personal interviews with each participant were conducted between October 2021 and September 2022 in nine different prison units, from the highest security level to transition prisons. The interviews lasted between 50 minutes and three hours and were audio recorded; they were based on a semi-structured interview guide that included questions from McAdams’ (2008) *The life story interview*. The topics covered important life events (e.g., high point, low point, turning point, and life challenges) as well as perspectives on the past, present, and future. The interview guide was used flexibly. To explore the recidivism process in detail, a set of questions focused on what the participants experienced during previous periods of imprisonment and what their lives looked like after release up until the point when they reoffended and were apprehended. Some questions probed explicitly into why they had reoffended and what they thought could have led to a different outcome.

Prior to data collection, I created a research journal that I continuously updated with the methodological and practical steps taken to gather my data. After every interview, I also wrote a short memo that reflected my immediate thoughts regarding how I had experienced the interview (setting, atmosphere, conversation flow, etc.). My memos often included notes on interaction during interview breaks, as well as some initial analytical thoughts and questions. I revisited and used these for critical review and/or confirmation of my interpretations during data analysis.

Conducting two interviews with each participant served several purposes (Read, 2018). First, given the many questions I wanted to ask, I was concerned that the interviews would be tiring and too time consuming. Second, due to the pronounced stigma attached to sexual offending, I presumed that it would take some time to build rapport and create an environment where participants were comfortable talking to me about their experiences. Third, as the purpose of this study was to gain deeper insight into a topic that is not well understood, I wanted to be able to transcribe and revisit the first interview before carrying out the second one in order to have a basis for further insight and follow-up questions. The second interview was conducted between 1.5 and 5 months after the first one, depending on the prisons’ preferences regarding visits. This time gap ensured that both the participants and I had a chance to reflect on our conversations. The second interview also served as a sort of member checking as I briefly recapped what we had been through in the first interview and gave the participants a chance to correct, add, and elaborate. I also asked them if they had any concerns after the first interview that they wanted us to address. Given the amount of information gathered, the complexity of the participants’ stories, and the narrative work that likely took place during (and between) our long talks, conducting multiple interviews made methodological sense.

### Data Analysis

The interviews were transcribed verbatim and coded with the software NVivo. As this is an exploratory study, the initial structuring and coding of the data was based on thematic analysis, which is a flexible method that seeks to understand the experiences and thoughts present in the data (Kiger & Varpio, 2020). The familiarization process involved reading the transcripts several times and sorting the information into main categories to get an overview of the data. I created codes that reflected the participants’ answers to the questions in McAdams’s life story interview and additional codes to sort what they said about treatment, self-image, recidivism prevention, etc. A brief timeline of each interviewee’s criminal history (including convictions, releases, and other relevant events) was drawn. I then reread all the transcripts, specifically searching for sections where the participants addressed their reoffending and/or where they tried to explain or make sense of it. Through thematic coding, three superordinate themes evolved. However, as these themes appeared in the same overall narratives, a more interpretative analysis followed. As narrative analysis is typically concerned with the combination of the content and the form of the language in a story (Sandberg, 2022), I developed codes to capture how certain narrative techniques or strategies were used to balance out the overall narrative. At each analytical step, I revisited the broader life stories of the participants to inform my interpretation of what they had said. The quotations presented in this article have been translated into English and edited for brevity and clarity.

### Ethics

This study was approved by the data protection officer (*Personvernombudet*) at Oslo University Hospital and the relevant correctional agencies. Each participant provided written informed consent, which was obtained either by the prison staff before the first interview or by me during the first face-to-face meeting. To ensure that the participants’ decision to participate was sufficiently informed, I explained my background as a former employee in The Correctional Services. I also told them about my current employment which has resulted in in-depth knowledge of the sexual offending treatment and research field, as well as many professionals involved in it. No incentive was offered for participation. The participants were assured that accepting or declining the invitation would not impact any aspect of their sentencing and that they could freely withdraw from further participation during or after the interviews without any consequences. Confidentiality and anonymity were strictly maintained throughout the research. Each interview was ended with a debrief to ensure that the participants were feeling well. None of the participants expressed discomfort or a need to talk to prison staff or a psychologist after the interviews.

## Results

### “Why did I Reoffend?” Making Use of Available Narratives

The thematic analysis focused on the causes of reoffending in the men’s recidivism accounts; to whom or what they attributed responsibility. Three superordinate themes evolved as they occurred and recurred across explanations and thus presented as the most commonly *available* narratives. While acknowledging some of the men’s explicit reluctance to use the word “blame” when talking about external causes, these themes center around three different recipients of blame. However, themes were not mutually exclusive but are meant to show where one might try to place responsibility for one’s failure in an overall recidivism narrative—on “the system,” the (hopeless) situation, or oneself.

#### Blaming the System

A narrative that frequently occurred in the explanations of recidivism can be described as “intervention inadequacy”. More than half of the participants explicitly or implicitly claimed that “the system”—the correctional services, health care services, or whichever professional agency was considered responsible for providing help to prevent future crime—could and should have done something during their previous sentences to reduce their risk of recidivism. Most often, the men focused on the lack of access to therapy or other opportunities to discuss their offending during prior periods of imprisonment, as well as on the absence of post-release support. For instance, Morten said, “It is only now, with this last sentence, that I have gotten the offer of getting help or treatment. Other than that, there’s been nothing.” While explaining their reoffending, some participants stressed that they really did not want to blame the system; still, they implicitly did so by expressing surprise and disappointment at the laissez-faire correctional approach they had experienced (Ievins & Mjåland, 2021). Adrian said, “I went to prison and was there for 2 years, and there was no follow-up either before or after. That surprised me a little”. His concerns were echoed by Isak, who had experienced a total lack of post-release support.

When I got out the second time, I had zero follow-up. I had no one to turn to. … I went to the GP; I applied for a psychologist, and not a single person wanted to take the case. No one. And then I was screwed, y’know. (Isak)

A few men more explicitly attributed responsibility for their reoffending to the system by saying that things would probably have been different if they had received proper support the last time they were convicted of a sexual offense.

[In this matter], Norway has a long way to go. When I served that sentence at [prison A], they had no kind of program, and no one talked about your sentence at all. It was kind of about just getting through those six months. I think that if I had had the opportunity that I got at [prison B], with the programs and all those things, the internet offenses wouldn’t have happened. And maybe the other things wouldn’t have happened either. (Richard)

Kristian said that if he had gotten help and someone to talk that “wouldn’t give up so easily”, things would likely have been “totally different”. He also explained why he had wanted a *forvaring* sentence thus:

My attorney and I talked about it [a *forvaring* sentence], and he said that there was a possibility that I would get it. He also told me a bit about what it was, y’know, and he said that among other things, I would be entitled to treatment or help. And the moment he said that, I said “I want that,” because then I could think that there’s hope for the future, that I won’t keep going in and out [of prison], y’know. (Kristian)

In summary, and in line with Harris’s (2017) findings, many of the men I spoke to indicated that they were clearly not ready for release on the previous occasion(s) when this had happened because, in their opinion, the system insufficiently prepared them for release.

#### Blaming the (Hopeless) Situation

A “narrative of hopelessness” also recurred in the explanations of many of the participants. To account for their recidivism, these men described how situational factors prior to their reoffending had made them “give up” or “simply not care about anything.” They painted a picture of a hopeless situation in which they had little to lose.

It’s like it was not even worth living [given] the way I felt. Even if I hadn’t ended up doing anything wrong, y’know. There was no joy in my life, nothing to look forward to. Like when my dad insisted that I should join a trip to our cabin or to a soccer game and things like that—I was kind of “Why would I do that?” Y’know. “I don’t enjoy it at all.” (Kristian)

The exact problems that led to this overall feeling of hopelessness were many and varied, but they mostly dealt with the men’s here-and-now situations after their previous releases—poor economic conditions, difficulties in finding work, loneliness, the loss of a loved one, broken relationships, or simply internal and external stress. Adrian was released right before the COVID-19 pandemic began. He described how, initially, he thought he would not reoffend, but then the circumstances worked against him.

Then it’s all the same again, y’know. You sit there without a job, and there’s a pandemic. So, you buy a computer because you’re bored, and you just end up staying inside drinking. And that doesn’t work very well. Then, I was kind of back in the same place. (Adrian)

William described how he felt he had been “released to nothing” after his previous conviction. His financial situation was poor, and he had to move back with his parents. The death of his mother was the last straw for him.

My mum got cancer and died. Then things escalated even more, because I’ve always had a good relationship with my mother, and that’s been one of the things that has held me back. … I also felt like the world is a shitty place and treats me badly, so I’m allowed to treat it badly. Then I took it out on [the victim], even though she had never done anything wrong. (William)

Sven was in what he described as a psychologically abusive relationship that made him feel subjugated, and he had no social network to support him. He explained how this situation contributed to his offending behavior with the following words: “I think that my cup was full. I couldn’t take it anymore. I had to somehow let it out. I think I couldn’t hold it inside anymore.” Sven also referred to his re-offense as a “cry for help”; he needed someone to see him and help him out of his hopeless situation. Several men in the sample echoed this kind of claim. Mikael explained how, upon his latest release, he had been deep in debt, including victim compensation claims following his conviction. He worked long hours to make ends meet and could not afford time off. In his view, this situation led to new offending behavior.

I’ve kind of landed on the conclusion that it really was a cry for help. … I’m not going to blame my economic situation and use that as an excuse for why I’m here, but the sum of it all—it was just too much, y’know. (Mikael)

In these excerpts of recidivism stories, an overall hopeless situation was portrayed as the problem. As was the case with the narrative of intervention inadequacy, these accounts externalized the responsibility for reoffending to something more or less beyond the men’s control.

#### Blaming Oneself

The third recurring narrative was that of self-blame. The explanations that made up this narrative often included descriptions of the absence of much-needed interventions; but when this was the case, the men explicitly blamed themselves for not seeking help. Many of the men in the sample repeated phrases such as “It was my fault” and “I know I can’t blame anyone but myself.” Several of them also claimed that they knew they needed help and were at risk of reoffending but that they were “too weak” and “too cowardly” to ask for support. Fredrik, for example, had this to say: “Had there been any initiative in me, I would have asked for help. But I didn’t.” Another participant, Adam, said the following:

[In prison,] the psychologist told me, “If you come across situations where you think like that, don’t hesitate to contact me.” And I was too cowardly to do that. Yeah. So, I let that family down. I especially let that boy [the victim] down. I let myself down, and I let everyone around me down. Yeah, I consider it a massive betrayal on my part. (Adam)

The narrative of self-blame was also evident when I asked William what he was trying to achieve with his offenses. He replied:

Well, God knows! Because… That too sounds foolish, but, um, the things I did with [the victim], they didn’t give me any joy. … But, at the same time, I didn’t have enough backbone to go back to the local psychiatry unit and get a new psychologist. I didn’t have enough backbone to stop things. … [I felt like] I didn’t do anything wrong; I was always in the gray zone. However, I pushed that gray zone and, in hindsight, I see that I knew for a long time that what I did was wrong. But I rationalized it by saying to myself “It’s not that bad.” (William)

Here, William blames himself and his rationalization of offending behavior. He implies that although he knew that what he was doing was wrong, he was too weak to do something about it. William was one of the men who had a poor self-image not only at the time of offending but in general. This was a common trait in the sample. Some participants attributed their negative views of the self to childhoods filled with trauma, including bullying (*n* = 10), physical abuse from caregivers (*n* = 4), and being subjected to sexual abuse (*n* = 5). These experiences were seldom used as explanations for their own offending, though this did occasionally happen. Others described “good” or “normal” childhoods, but they added that they had “always” felt insecure and had a negative self-image. Although many participants had managed to improve their self-images and now looked differently at themselves, this problem was part of their recidivism narrative. Some presented it as the main cause of their crimes. For example, during the sum-up of my final interview with Kristian, he attempted to draw some conclusions regarding the many possible causes we had discussed in our conversation. This is what he said:

I think it’s just my self-image, and kind of the self-hatred and contempt and just… I had such a nice life, y’know. And I kind of ruined it myself; there’s no one else to blame. Only myself, and how stupid I’ve been. … I never felt that I was good enough at anything, y’know, but at least I have no one to blame [he laughs briefly]. (Kristian)

Some of the men in the sample blamed themselves occasionally, and others did so routinely; however, their stories were far from unequivocal. The next section shows how constant battles between internal and external causes, as well as between taking some or full responsibility, were present in the narratives.

### Searching for the “Acceptable” Recidivism Story

**Figure 1.**
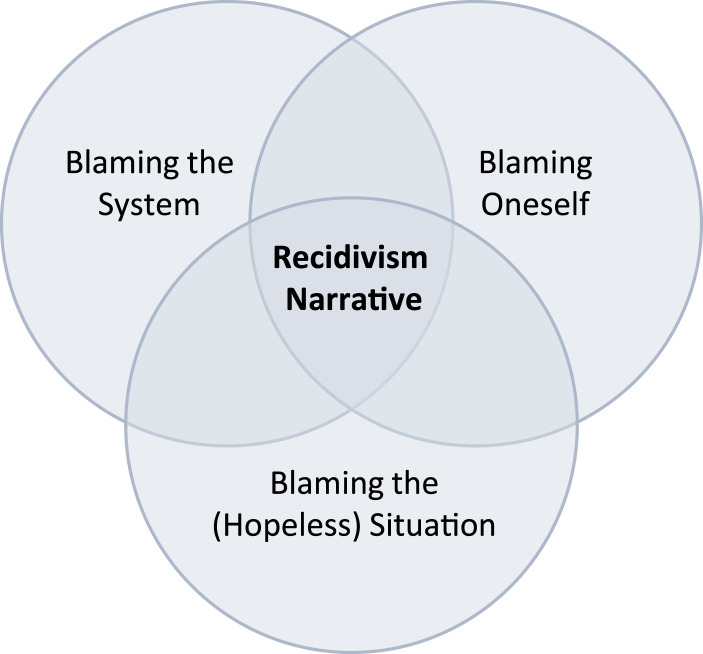
Overlapping Explanations for Recidivism.

Despite the three described superordinate themes that manifested across stories, none of the participants gave a short and simple answer as to why they had sexually reoffended. As a result, it did not make sense to divide the men in this sample into distinct groups according to where they put most of the blame for their recidivism. On the contrary, their narratives overlapped, as demonstrated in Figure 1. This section attempts to demonstrate why explaining sexual recidivism is so challenging.

Many of the men underscored how they truly wanted to increase their own and others’ understanding of their reoffending. At the same time, they clearly stated that they found explaining it a difficult or even impossible task. Two of the men explained this challenge thus:

Well, there are still many things, y’know, that I kind of don’t have an answer to or have not managed to understand. Yet. … I really want an answer but I might never get one, other than the fact that there are many factors. (Robert)

I can understand that it’s difficult for people to understand why we do the things we do, but honestly, we don’t always understand it ourselves. And it’s not, um, it’s not to distance ourselves from what we’ve done; it’s actually because we don’t know. So, the worst question I get is, “Why did you do it?” I think that’s a stupid question because if I knew the answer, I might not have done it. (Mikael)

Despite this general claim that it was more or less impossible to answer why they had sexually reoffended, the participants presented several possible causes throughout our conversations. However, they seldom pointed to a single decisive factor, and many of the suggested explanations were followed by phrases such as “But I don’t know” and “This is of course just one possible interpretation.” Toward the end of the interviews, I asked the participants how they had experienced being interviewed about this topic and whether they felt that they had had the opportunity to tell me about the things that mattered to them in their recidivism stories. I also reminded them of the purpose of my study—to understand *their* perspectives and accounts of why they had reoffended. These final exchanges tended to be quite long and often led to increased ambiguity and ambivalence. The fact that most of the men lacked a natural ending to their story—a conclusion—seemed to bother them. Many said that they felt unsure about whether they had managed to give me “the answers I needed,” and they expressed concern that they might have left out important details or not given me a coherent account. Apparently, explaining sexual *re*offending required more effort than explaining sexual offending per se. This became clear when I asked Adrian if he could tell me about an important turning point in his life. This is what he replied:

Yeah, well, I think the greatest turning point I’ve had was now, just before this [sentence], in terms of how I look at myself. Because the last time, I kind of managed to write it off as a one-time event, um, but it turns out that it’s not. … When I got out the last time, I thought that it was gonna go well and that I could just go on with my life. … Then, when I got out and kind of continued, I realized that there was something, um, there’s something I need to do something about. (Adrian)

Thus, while a single incident of sexual offending allows for the possibility of dismissing it as a one-time event, explaining why one has repeated this behavior requires a different justification. It is simply harder to make sense of reoffending.

The participants often presented multiple and even contradictory explanations in an attempt to understand and narrate why they had reoffended. Some of them also included our interviews in this process of meaning making. At the end of my final interview with Lukas, he explained how our conversations had made him think more about his need for closeness as a contributing factor in his recidivism and, possibly, even as a legitimate *reason* for his many incidents of reoffending. He said that “one usually winds up following one track” in an attempt to clarify and that, up until that moment, he had “mostly followed the track of self-medication,” where his crimes against young girls were a result of poor self-image, low self-esteem, and a need to feel better whenever he felt bad. “I’m not 100% sure, but to me, that sounds like a good explanation for what I’ve done, or the prelude to what I’ve done,” he said. As a response to his reasoning, I made a comment that there likely were “many pieces to that puzzle.” Lukas followed up by sharing his thoughts on how a narrative might not only be in the making but also subject to external influence.

[T]hat’s why I’m very excited to kind of see what you get out of this project, because I’m very unsure of the answers myself. I try to give good answers, um, and I assume that others are trying to answer the best they can as well. And, my experience is kind of that, in the [therapy] group as well, many [of us] fumble for the answers, and some even try to give the answers that they believe others want to hear. (Lukas)

**Figure 2.**
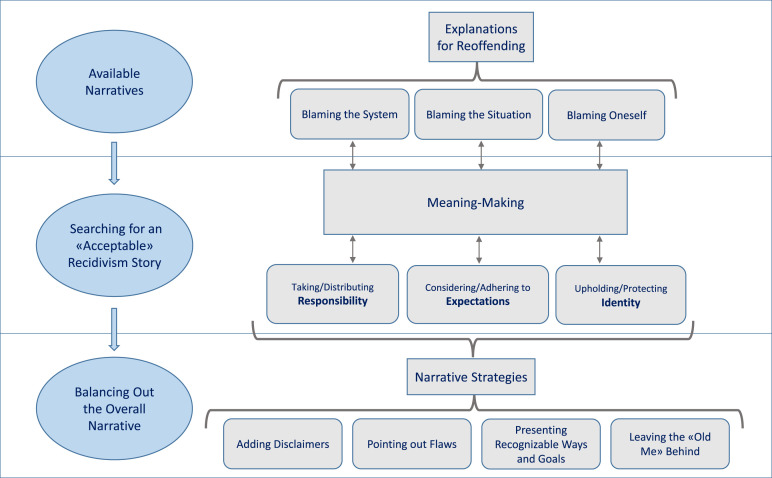
Explaining the Unexplainable. Thematic and Analytical Map.

Lukas’s words translate into questions that the men in this study needed to consider. What is a “good” answer to why one has committed repeated sexual crimes? How does one work toward a narrative that meets both one’s own and others’ expectations of what constitutes an “acceptable” story? The participants dealt with this narrative problem in different ways, as demonstrated in Figure 2. Below, I present four observed strategies.

### Narrative Strategies: Balancing Out the Overall Story

#### Adding a Disclaimer

The first strategy consisted of a sudden change in the narrative achieved by adding a “disclaimer” toward the end of a sequence that involved an attempt to explain reoffending. This disclaimer seemed to serve the purpose of increasing the story’s complexity through de-emphasizing the importance of the most recent argument and preparing for the introduction of another explanation as the story continued. When the participants realized that a sequence of the overall narrative attributed most of the blame to them, they often reached for an external factor that could take some of the responsibility.

I can’t blame anyone or anything but myself because I’m the one who has done it, but I have a feeling that… I feel like I’m smart enough to… Had there been an offer for help, y’know, or had I been given the opportunity back then, I think things would have stopped there. (Richard)

However, disclaimers were also used to emphasize, or “adjust up,” internal responsibility or agency after attributing responsibility to external factors, thus verbally holding oneself accountable. This type of disclaimer, which shifted the narrative from the externalization to the internalization of responsibility (rather than the other way around), was more common because the men were generally very reluctant to conclude that someone or something else was *fully* responsible for their reoffending.

Well, if I’m going to put some blame on the Correctional Service, it has to be when I asked for help in prison. Even if it was a trivial case, and even if the sentence was too short for me to… They should have referred me to the local psychiatry unit, and they should at least have sent a report of concern … to both the local police office and the local psychiatry unit and that kind of thing but… I guess I just have to say that it’s my own fault. (William)

The above narrative sequence starts out as a typical example of blaming the system for its inadequacy and then takes a sudden turn toward self-blame. Many stories went back and forth between the three observed superordinate narrative themes, and disclaimers frequently appeared as part of this process. Hence, although swapping explanations might seem ambiguous and perhaps even contradictory at first, this narrative strategy served an important function: balancing the distribution of internal and external responsibility to meet others’ expectations. These others could be the researcher, those who will read the study’s results, or a more general imaginary audience. Nevertheless, disclaimers seemingly served as tools to influence others’ perceptions of the participants and their stories as well as (perhaps) their own.

#### Pointing Out Flaws

Another strategy used was to actively point out the flaws in their explanations—that is, the things they said that they knew they were not supposed to say. The participants likely assumed that I shared some of the mainstream perceptions of people who have sexually offended (Zatkin et al., 2021) and would thus disregard their stories as unrealistic or dishonest. This is not just an assumption; some of them actually told me so. At the end of my second interview with Jens, he said that he hoped he had managed to answer my questions but that he had found it difficult because he usually assumed that people perceived his explanations as minimizations of harm or attempts to explain away what had really happened. He ended this argument by saying, “I imagine that you also think this when you hear this version of what happened.”

Many other participants were also very aware that their attempts to account for their reoffending would be taken as excuses or examples of how they employed cognitive distortions regarding their criminal behavior (Friestad, 2012). Some said that there were certain ways of reasoning they could never present in court or during a risk assessment. They offered these thoughts in our interviews, but they pointed them out as flaws by making comments such as “If I had said that, it would have been considered a cognitive distortion” (Isak) or “Of course, I’ve later learned that that’s a cognitive distortion” (Mikael). The men also occasionally paused in the middle of an explanation just to add phrases such as “I know how this sounds” or “I guess I’m minimizing again now.” Sebastian was convicted of several rapes committed while he was drunk. At the time of the offending, he did not feel good about himself. He explained how he harbored a lot of anger due to childhood trauma and had been drinking heavily for some time. I asked him whether, before the sexual assaults, he knew that he was capable of committing violent crimes and that things were going downhill. He replied: “Yes, but I still didn’t stop. Um, aaah, it sounds like fucking excuses, y’know. But, um, I just kept drinking.” I asked him what he thought could have stopped him back then, and he said,

What stopped me was that I got caught, and I’m grateful for that. … It had gone way too far many times. … And … it’s not rocket science that alcohol has been a strong, um, contributor, um… That’s not the right word either, but it has been a great part of it. So, that’s kind of the first priority—get rid of that part, take away the alcohol. Um, oh, it sounds like I’m making excuses, and that bothers me. It bothers me a lot. (Sebastian)

When I commented that what he had said had not bothered me because “I do not think like that,” he simply replied, “No. But *I* do.” There appeared to be a conflict between what Sebastian might have *wanted* to say, what he knew he *should* have said, and how different versions of the story would shape my view of him (or others’ views). Therefore, pointing out these flaws seemed to function as a tool to deal with the “narrative discomfort” the men experienced when their stories did not add up or adhere to expectations. When participants repeatedly interrupted their reasoning like this, I sometimes made comments such as “You don’t have to say that anymore, I get it.” I also tried to eliminate these excuses for excuses (!) from the narratives by repeating that I was a researcher, not a treatment provider, judge, or risk assessor. I stressed that I was interested in *their* reasoning and their honest thoughts about why they had reoffended. The fact that I did not get very far in this respect highlights how the need to consider others’ perspectives while telling their stories was deeply embedded in these men.

#### Presenting Recognizable Ways and Goals

The participants also presented thoughts about what they tried to achieve with their offending. Only rarely did someone say that their crimes were purely instrumental means to achieve sexual satisfaction or an adrenaline kick. Most of the time, the participants’ reflections pointed to an attempt to achieve some kind of relationship or sense of community, as well as a feeling of being worth something. These are goods that are considered universally human; thus, the implication was that they applied also to them (Harris et al., 2019; Ward & Stewart, 2003). Although assessing “primary human goods” was beyond the scope of this study, an important observation here is that these men presented explanations that can easily be translated to The Good Lives Model framework (see Ward & Stewart, 2003). For instance, Lukas described his sexual chatting with children as “kind of an arena where I have felt comfortable with finding girls or forming some type of relationship, you know; either a sexual or a romantic relationship.” Richard saw his offenses as a response to his unmet need for attention and recognition: “I think it’s mostly about being seen and liked. That someone kind of cares about me. I didn’t think like that back then, but I see it now.”

By presenting their offenses as a means to fulfill needs that are important and recognizable to most people, the men in the sample managed to show their inherent human traits. Perhaps unintentionally, this strategy served the purpose of conveying the important notion of “I’m very much like you.” The final strategy below underscores the need to create and uphold such a self-narrative, as it involves distancing oneself from who they were “back then.”

#### Leaving the “Old Me” Behind

This strategy consisted of separating the “me” that sexually offended from the person one truly is (see also Presser, 2004). Although this strategy often manifested itself when looking forward, it was also present in the direct accounts of the participants’ reoffending. When explaining what led to his re-offenses, Richard described how he used to sit alone in his apartment, scared and depressed, with the curtains closed. He had severe problems with his psychologically and physically abusive ex-partner, whom he feared. Starting from the narrative of hopelessness, he said that his reoffending began because he sought comfort in online chat rooms. He then started to pressure minors to send him sexualized material. He elucidated how that could happen thus:

Yeah, I became the guy who pushed them, y’know, um, instead of being the person I usually would have been, the kind one and things like that. … I didn’t care who I was talking to [online], what I wrote, or anything. My life was ruined. My life was gone anyway, so I didn’t care. (Richard)

In our next interview, I presented to him my recollection of what he had said about becoming the guy who pushed, and I underscored what I saw as the main point—he is not *really* that type of person. I asked him if he could talk a bit more about whom he was and how being someone who pushed did not fit his view of self. He elaborated on these aspects with the following words:

[I]’ve always had a desire to [help], um, like my mum has said to me, “Cut it out, you can’t save the world all by yourself.” I used to be like that, very keen to help. … And then, when I sit down [in front of the computer], I forget that part. I forget the helping part, y’know, I kind of turn more toward, “How do *I* feel? What am *I* thinking? What do *I* want?” Um, instead of thinking about whether there’s any help in that. So yeah, that was kind of the greatest change—that I stopped being the kind, helping one and just didn’t give a damn. (Richard)

Although the causal relationship between Richard’s situation and his offending is unclear in his account, this part of the story tells us something about him as a person and who he really is and always has been—someone who is kind and caring. The situation he found himself in, however, made him forget that. A similar interpretation can be applied to Adam’s statement that “some kind of moral filter was turned off” when he committed his crimes. This statement implies that, deep down, he is a moral being and that offending does not fit his “real me” (Maruna, 2001).

Many of the participants’ narratives included groundbreaking identity change, which distanced the men’s present selves from their “offending selves.”

[Prior to my second conviction] I was so far down, both physically and mentally, that I, um… I was just waiting to get caught, because I knew I would get caught in the end, and I needed help. Yeah. And then, from that summer in [year], my life changed, because I started a program in prison, and it totally changed me. I got to talk about my feelings, my thoughts, and why I had committed the offenses, and everything just fell into place. (Sven)

Unfortunately, I also have a couple of violent offenses and robberies—stuff like that—on my record, so it’s kind of… Yeah, I’ve been out of balance, to put it that way. But, um, seeing a psychologist, which I’ve done, and making use of the prison’s programs and follow-ups, I see that as kind of the great turning point in my life. So, I guess I’ll call it a 2.0 version of myself, and it’s clear to see. Very clear. (Robert)

These quotes highlight how the men considered the availability of arenas to talk about their offending while imprisoned as crucial to help them progress. In some of the overall narratives, looking toward the future while stressing identity change also became a powerful tool.

[T]he time in prison has kind of changed me. Before, I used to be super selfish; I only cared about myself. I could yell at my wife, y’know, at a party or even if I’d only been sitting at home drinking by myself, and the next day I just thought, “New day, new possibilities—a clean slate,” and what had happened the day before was forgotten. No apology. Nothing. That was me in a nutshell. Zero empathy. Nothing. … When you’ve been inside for as many years as I have, you’ve reviewed yourself quite a few times. …. There are things I can’t change—what’s done is done—I can never change it. It will follow me for the rest of my life, but, um, that doesn’t mean I have to be a bad person in the future. (Tor)

These are examples of how one can show adherence to the expectation of taking responsibility by blaming oneself—but one’s *old* self, which has now been left behind.

## Discussion

This study explored the narratives that Norwegian men who have sexually reoffended provided to explain their experiences of release and recidivism. Three main narrative themes emerged from the stories of the 16 men included in this study: blaming the system, blaming the (hopeless) situation, and blaming oneself. The first two represent versions of Sykes and Matza’s (1957) “denial of responsibility,” where one claims that one’s behaviors are accidental or due to forces beyond one’s control. In the latter, the attribution of responsibility shifts from external to internal factors. However, results also showed that the participants had a difficult time making sense of their recidivism, especially when trying to present it as a coherent and “acceptable” story. Themes overlapped as the participants presented several ambiguous explanations of their sexual recidivism. They were generally reluctant to reach conclusions regarding why they had not managed to stop their offending after their last release and frequently used disclaimers to uphold a form of narrative complexity. Sandberg et al. (2015) have argued that this “plurivocality” is often missed when stories of violence are described in established criminological traditions. In this study, however, it has emerged as a main finding. Storytelling serves several functions, as illustrated by the participants’ frequent use of narrative strategies to balance responsibility, expectations, and identity in their overall narratives. The men’s attempts to please an imaginary audience while upholding a moral identity are thus central to the following discussion.

Despite the narrative analysis conducted in this study, however, I do not wish to dismiss the importance of what the participants said concerning the inadequacy of interventions. The men asked for arenas to talk about not only their offending but also their wider lives and experiences, both during imprisonment and after release. More than half of the interviewees said that they had needed “someone to talk to” in the past but that they had had few realistic options to do so. They seemed frustrated that therapeutic settings had not appeared as truly accessible until they had reoffended. As a result, many of them had the impression that their previous sexual crimes had not been taken seriously by the system and that prior sentencing had not been sufficiently meaningful (Ievins & Mjåland, 2021). Many of these men probably did not pose a statistically high risk of recidivism at the time. Moreover, their risk might not have been assessed due to relatively short sentences or simply the fact that the prison did not offer sexual offending-specific psychological treatment. Thus, they might not have been identified as being in particular need of intensive intervention or treatment (RNR principles). However, risk prediction is not an exact science, and there is a continuum of possibilities (beyond imprisonment) from zero intervention to intensive specialized treatment. Several participants claimed that they had *now* received the support they needed, and many spoke warmly of the prison staff and treatment providers who had helped them change. These men shared the assumption that it was helpful to have someone to talk with openly who would not judge them, not use excuses against them, and not criticize their “distorted thinking” in this process.

### Explaining the Unexplainable

Given the moral condemnation of sexual crimes and those who commit them (Harper et al., 2017), the starting point of the participants’ stories was challenging. The media, the prison environment, and social interactions reminded these men of the culturally available narratives and mainstream perceptions surrounding people “like them.” Thus, getting a fair chance to prove one’s ability to abstain from offending could not be taken for granted upon release. Most people released from a sexual offense conviction do not reoffend (Hanson & Bussière, 1998; Hanson & Morton-Bourgon, 2005; Lussier et al., 2023). However, the men in this study squandered their second chances by committing another sexual crime, which proved that their deviant behaviors were *not* one-time events. For this reason, they might have expected to be considered less trustworthy (Ugelvik, 2021) or that their explanations and overall stories would be considered by their audience to be less palatable. Although some of the men might not have seen the causes of their sexual offending and *re*offending as substantially different, the frames and contexts surrounding their stories most definitely were. As incarcerated individuals who had already failed, they were well aware of the expectations to take responsibility for their actions, get rid of excuses, and change their mindsets. Thus, their narratives needed to be constructed in a way that dealt with these expectations in terms of what a “good” explanation looks like. The analysis identified clear attempts to adhere to these social expectations in the narratives, although doing so was not straightforward. Rather, it was clear that the men experienced discomfort in their attempts to place blame, also when they chose to blame themselves, as implied by the use of the different narrative strategies. Hence, there seems to be more to this overall story in terms of fundamental identity work.

To be able to rewrite a shameful past, one needs to develop a story that can explain the past and give a convincing account of why the present is different (Maruna, 2001; McAlinden et al., 2017). Few people, and none in the current study, wish to be seen simply as an irredeemable “sex offender”. Therefore, for most, the question “Who am I?” is important when trying to answer the question “Why did I offend?, as one needs to explain how one could do the things one did and still not be a bad person. In order to make meaning, people need to “go beyond the plots and event details of their personal stories to articulate what they believe their stories say about who they are” (McAdams & McLean, 2013, p. 236). The findings of this study strongly indicate that the participants found themselves in a typical catch-22 situation when trying to account for their most recent failures. As noted by Maruna and Mann (2006) for incarcerated individuals *generally*,

if they make excuses for what they did, they are deemed to be criminal types who engage in criminal thinking. If, however, they were to take full responsibility for their offenses – claiming they committed some awful offense purely “because they wanted to” and because this is “the type of person” they are – then they are, by definition, criminal types as well. (p. 158)

The severity of the offenses carried out by the men in this study and the fact that they had repeated them made the task of telling the story more demanding than explaining just any criminal incident. Due to the nature of their offenses, the need to shift responsibility for their behaviors from themselves to something or someone else, might have seemed more pronounced as yielding to the complete internalization of responsibility would mean staining their identities beyond repair. At the same time, the opportunity to blaming others without being labeled irreversibly deviant or cognitively distorted might be equally small again due to the nature of the offenses. In other words, the participants seemed to be in urgent need of using, but deprived of, the tool commonly employed to account for failure while upholding a positive identity—that is, the externalization of responsibility. The narrative strategies observed in their explanations represented their attempts to fix this catch-22 situation by protecting their identities while adhering to societal expectations and a perceived obligation to assume at least some responsibility. The result was a form of narrative ambiguity that could be mistaken for something exclusively negative and a lack of insight. Interpreted in a more positive manner, however, the ambiguous stories presented in this article might reflect an attempt at meaning-making and a legitimate desire to change. While the “acceptable” sexual recidivism story is perhaps non-existing, the occasional externalization of responsibility might simply be an aspect of the best explanations these men could offer within the constraints of their present conditions, and not an attempt at absolving themselves of responsibility.

### Recidivating Desisters?

We cannot know whether the men interviewed in this study have committed their last sexual offenses. However, the stories presented by many of them could easily be mistaken as desisters’ narratives; they often had the character of what Maruna (2001) referred to as a “redemption script.” Some of the participants emphasized that even when they were “at their worst” and committed their re-offense, “deep down” they were good people (Maruna, 2001, p. 89). Thus, if narratives are indeed constitutive of future behaviors, asking them to set this thought aside seems counterproductive. In contrast, it is highly relevant to accept the importance of the identity work involved in exploring different explanations while trying to balance the overall narrative. The factors the men presented as contributing to their reoffending were many, complex, and unclear. For this reason, the participants may benefit from arenas where they can talk about their experiences and be supported in their meaning-making processes, both before and after release, regardless of the lengths of their sentences. That way, they may be better equipped to live out pro-social narratives in the future.

### Methodological Reflections

Readers must keep in mind that participation in this study was voluntary, and the stories presented here are the stories of those who chose to talk about their reoffending. Moreover, the vast literature on reflexivity reminds qualitative researchers that influencing the setting, the participants, and the interviews is almost inevitable (e.g., Blagden & Pemberton, 2010; Olmos-Vega et al., 2023). While this is not a limitation in and of itself, being aware of this issue is important, also when it comes to interpretation and dissemination of results. In the study’s interviews, I represented another human being to talk to. In terms of positionality statement, I also came with a mindset shaped by having spent years working in the Correctional Service and the broader field of sexual abuse research. I was open about my professional background and the fact that I had worked closely with treatment providers in the field, and this undoubtedly shaped the conversations I had with the men. Hopefully, however, this also gave the participants a sense of mutual trust and convinced them that the interviews were a safe space and that I would attempt to understand them rather than judge them as “bad people” simply because of their criminal histories. Hence, given the issues related to creating a safe environment and building rapport (Blagden & Pemberton, 2010), my background (and the men’s knowledge about it) might as well have been an important strength. I managed to include participants from a population whose narratives have so far been understudied, and the repeated interviews strengthened the research design by providing rich data suitable for exploring the many quirks found in “the stories we live by” (McAdams, 1993). Finally, as with most interview-based qualitative research, and particularly in the study of narratives, the final presentation of the results is my interpretation of what the participants wanted to communicate. While I have strived to adequately reproduce their voices, analyzing qualitative data undoubtedly involves considering what stands out as most interesting and relevant (Copes et al., 2016).

### Conclusions and Future Directions

A narrative or story does not evolve in a vacuum; it is dependent on the social and cultural context in which it is told. This study investigated the recidivism narratives of a sample of men incarcerated for a sexual offense in Norway. In countries where laws, penal practice and interventions look different, other narratives may seem more available to those who have failed, especially in regards of whom or what to blame. However, interventions in the sexual offending field tend to build upon common principles derived from a growing international base of clinical and scientific knowledge and their overall purpose is universal: to prevent future sexual offending. I have argued that this involves helping individuals in constructing and living out pro-social narratives. This study’s results might therefore be highly relevant beyond the Nordic “exceptional” context.

The findings of this study imply that attempts to explain failure should be taken seriously regardless of how ambiguous, ambivalent, constructed, or contradictory they may seem. The stories presented in such ways represent healthy expressions of potential to change. The fact that the men put so much effort into narrating their stories in a way that could adhere to others’ expectations shows that they cared about how others viewed them. Moreover, the fact that years of treatment, risk assessments, and living with the “sex offender” label continued to disrupt the men’s narratives is something that treatment providers and correctional staff can take advantage of. In terms of practical implications, perhaps one way to approach these ambiguous stories is as desistance narratives in the making (Maruna, 2001) that can be used as powerful tools in rehabilitation work.

The process of leaving the “old me” behind is central to a desistance narrative. To assist persons with criminal histories in establishing this process, guiding principles from narrative therapy might be helpful. Most importantly, however, we must provide hope, and safe spaces where they can work toward a desistant identity by narratively exploring who they are and who they want to be in the future. These spaces can be found beyond psychological treatment settings, in the daily interactions between convicted individuals and prison staff. Hence, even a short prison sentence represents an excellent opportunity to influence the self-narratives of those individuals who have failed in the past and assist them in their attempts to move forward.
